# Metformin Ameliorates Cognitive Deficits and Neuroinflammation in a Mouse Model of Familial Hypercholesterolemia

**DOI:** 10.1007/s11064-025-04658-7

**Published:** 2026-01-24

**Authors:** Natália Baltazar do Nascimento, Hémelin Resende Farias, Tainá Schons, Alex Paulo Zeferino Padilha, Mariana Viana Costa, Ariadni Mesquita Peres, Lucas dos Santos da Silva, Ricardo Maia Dantas, Jessica Marques Obelar Ramos, Matheus Scarpatto Rodrigues, Fernanda Telles, Fátima Theresinha Costa Rodrigues Guma, José Cláudio Fonseca Moreira, Rachel Krolow Santos Silva Bast, Andreza Fabro de Bem, Jade de Oliveira

**Affiliations:** 1 Department of Biochemistry, Basic Health Sciences Institute, Federal University of Rio Grande do Sul, Porto Alegre, Rio Grande do Sul Brazil; 2 Department of Psychiatry, University of Pittsburgh, Pittsburgh, PA USA; 3 Laboratory of Bioenergetics and Metabolism, University of Brasília, Brasilia, Federal District Brazil; 4 Brazilian National Institute of Science and Technology on Neuroimmunomodulation, Oswaldo Cruz Foundation, Rio de Janeiro, Rio de Janeiro Brazil

**Keywords:** Familial hypercholesterolemia, Metformin, LDLr^−/−^ mice, Behavioral alterations, Astrocytes

## Abstract

Familial hypercholesterolemia (FH), caused by mutations in the low-density lipoprotein receptor (LDLr) gene, has been increasingly associated with neurodegenerative and mood disorders. Studies with LDLR knockout mice (LDLr^−/−^) showed that neuroinflammation is a key event in FH-related brain dysfunction. Because mTOR inhibition has been shown to mitigate brain alterations in this model, we hypothesized that metformin, a drug reported to influence cellular energy metabolism, could attenuate FH-associated brain changes. To test this, adult LDLr^−/−^ mice received daily oral doses of metformin (200 mg/Kg) or vehicle for 30 days. During the final week, behavioral assessments were conducted, including the open-field test, novel object recognition and object reallocation tasks, and the tail suspension test (depressive-like behavior). Body weight, total cholesterol and glucose plasma levels were analyzed. Hippocampal astrocyte and microglial density, as well as the expression of genes related to neuroinflammation and synaptic plasticity, were evaluated. Metformin did not alter total cholesterol levels but significantly improved cognitive performance and reduced depressive-like behavior. The treatment also attenuated hippocampal astrogliosis without affecting microglial reactivity. Molecular analysis revealed reduced hippocampal TGF-β gene expression and increased PSD-95 gene expression and protein content in metformin-treated LDLr^−/−^ mice. Although a slight, non-significant reduction in the phosphorylated-to-total mTOR ratio was detected, no clear evidence of AMPK/mTOR pathway modulation was observed. Overall, metformin improved memory function and astrocyte reactivity in LDLr^−/−^ mice independently of cholesterol reduction and without demonstrable involvement of the AMPK/mTOR pathway, suggesting its potential as a therapeutic strategy for FH-associated brain dysfunction.

## Introduction

In the past few decades, hypercholesterolemia, a well-known risk factor for cardiovascular disease, has also been considered a causative factor for dementia and mood disorders [1–6]. In particular, familial hypercholesterolemia (FH) patients aged between 18 and 40 years display alterations in their neuropsychological performance [7], while middle-aged FH individuals have an increased incidence of mild cognitive impairment (MCI) [8]. FH is caused by a mutation in the gene encoding the low-density lipoprotein (LDL) receptor, which results in high levels of LDL cholesterol and premature cardiovascular disease [9, 10].

Our research group, using LDL receptor knockout (LDLr^−/−^) mice, corroborated the clinical data from FH patients [11–13]. These hypercholesterolemic mice exhibit memory impairments and depressive-like behavior at 3 months of age, accompanied by blood–brain barrier (BBB) disruption in the hippocampus and prefrontal cortex and neuroinflammation characterized by astrogliosis and microgliosis [11, 12, 14, 15]. Additionally, mTOR signaling has been implicated in the brain dysfunction induced by FH. As a proof-of-concept, treatment of LDLr^−/−^ mice with rapamycin, a classical mTOR inhibitor, improved neurovascular function and cognition in this model [16].

Another way to attenuate mTOR signaling is through AMPK activation [17–19]. In this context, metformin is of particular interest because it can activate AMPK signaling [20]. Metformin is a widely used anti-diabetic medication with neuroprotective effects, primarily through the modulation of neuroinflammation [21]. Rabieipoor and collaborators (2023) reported that a two-week treatment with metformin restored cognitive dysfunction in an experimental model of sporadic Alzheimer’s disease (AD), accompanied by improved astrocyte density and prevention of neuronal loss [22]. In aged mice, metformin attenuated cognitive decline, reduced hippocampal microglial activation and astrocyte hypertrophy, and reduced levels of proinflammatory factors, accompanied by AMPK activation and mTORC inhibition [23]. Moreover, in LDLr^−/−^ mice, metformin treatment prevented the formation of aortic atherosclerotic plaques [24].

Therefore, we hypothesized that metformin could attenuate the behavioral alterations associated with FH. To test this, LDLr^−/−^ mice were treated with metformin for 30 days.

## Materials and Methods

### Animals

Male 3–5-month-old C57BL/6 LDLr^−/−^ mice weighing 20–30 g from the Department of Biochemistry at Federal University of Rio Grande do Sul (UFRGS) were used in this study (*n* = 42). Mouse progenitors were obtained from Jackson Laboratories. The mice were allocated into groups of 5 per housing box (42 × 34 × 17 cm), under controlled temperature (22 ± 1 °C) and light (light-dark cycle of 12 h, lights on from 7 AM to 7 PM), with *ad libitum* access to water and standard chow. The UFRGS Ethics Committee approved the present study (Protocol #41034).

### Experimental Protocol and Pharmacological Treatment

Metformin (200 mg/Kg) [25] was administered by gavage daily to the mice for 30 days. The vehicle used was a sodium chloride solution (0.9% in distilled water). The dose and duration of the metformin treatment were selected based on protocols from prior studies investigating its neuroprotective effects in rodent models of cognitive impairment. Specifically, our treatment strategy is consistent with the methodology used by Farr et al. (2019), who demonstrated that 200 mg/Kg of metformin effectively improved learning and memory in the SAMP8 mouse model of Alzheimer’s disease [25]. Further supporting this approach, similar treatment parameters were shown to be effective in alleviating Alzheimer’s-like pathology in other rodent models [26]. The control group received the vehicle solution at the same frequency and period as the metformin group. Both treatment solutions were stored at 2–8 °C. The animals were randomized into two experimental groups (*n* = 19–24 per group) according to treatment: LDLr^−/−^ mice treated with the vehicle and LDLr^−/−^ mice treated with metformin. From the first day of treatment, animals were weighed weekly until the end of the experimental protocol. Capillary blood glucose was measured on day 0 and day 30. Over the last four days of treatment, the mice underwent the following behavioral tests: open field (OF), novel object recognition (NOR), object reallocation (OR), and tail suspension test (TST), to evaluate locomotor activity, hippocampal-linked memory, and a depressive-like phenotype, respectively. Following the behavioral tests, some of the animals were fasted for 12 h and then anesthetized via intraperitoneal injection with a mixture of xylazine (10 mg/Kg) and ketamine (80 mg/Kg). Blood was collected by cardiac puncture to investigate total cholesterol, and the hippocampus was dissected for Western blotting and RT-qPCR analysis. The remaining mice were anesthetized and perfused with 4% paraformaldehyde, and then brain tissue was collected for immunofluorescence assays. It is important to note that we conducted two independent treatment cohorts, each consisting of the two experimental groups previously described. Both cohorts were submitted to the same experimental protocol, with standardized housing, handling, treatment schedules, and assessments. Data from both cohorts were pooled for the final statistical analyses.

Given that the primary aim of this study was to evaluate the therapeutic potential of metformin within the established pathological context of FH, experiments were conducted exclusively in LDLr^−/−^ mice treated with vehicle or metformin, without the inclusion of wild-type (WT) controls. It is important to mention that the baseline phenotypic differences between WT and LDLr^−/−^ animals have been extensively characterized by our group in previous publications. Our studies consistently demonstrate that LDLr^−/−^ mice exhibit spatial memory impairments, astroglial activation, increased blood–brain barrier permeability, oxidative imbalance [14], age-dependent cognitive deficits and neuronal apoptosis [12, 27], memory impairment regardless of diet [12], as well as microglial alterations and changes in synaptic and tight junction proteins [15]. This approach also aligns with the 3Rs principles, reducing animal use without compromising the validity of the findings.

**Fig. 1:**
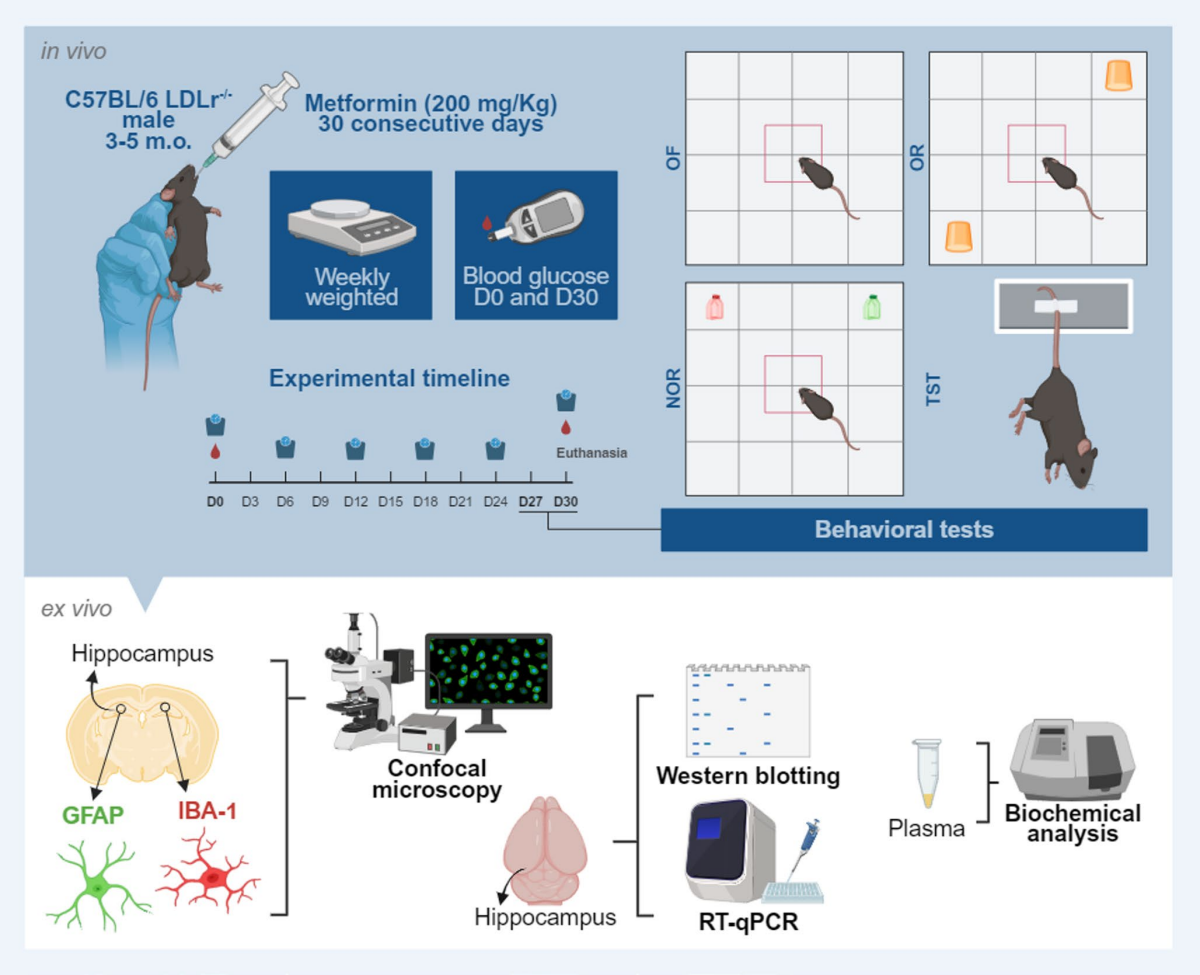
Experimental protocol. 3–5-month-old male C57BL/6 LDLr^−/−^ mice received vehicle or 200 mg/Kg of Metformin via gavage for 30 days. All animals were weighed weekly and had capillary blood glucose assessed on days 0 and 30 of the experimental protocol. Over the last four days of treatment, mice were tested for locomotor activity (OF), short-term memory (NOR), spatial reference memory (OR), and a depressive-like phenotype (TST). After euthanasia, brain tissue was dissected for immunohistochemistry assays, RT-qPCR, and Western blotting. Total cholesterol was determined from plasma

The experimental design is illustrated in Fig. 1 below.

### Behavioral Tests

#### OF

The OF test was conducted on the 27th day of treatment. The open-field test investigated locomotor and exploratory activity [28]. The apparatus consists of a grey acrylic box (30 × 40 × 40 cm) with the floor delimited into 9 quadrants. Each mouse was placed in the center square of the arena and allowed to explore for a 5-minute training period. To evaluate locomotor and exploratory activity, the number of crossings and rearings was recorded in a 5-minute test session.

#### NOR

On the 28th day of treatment, animals were subjected to the NOR test to evaluate their short-term memory. In this test, mice were exposed to 2 identical objects, and after a short interval, were re-exposed to 1 familiar object and 1 novel object [29]. The test was conducted in the same apparatus used in the OF, in 2 sessions of 5 min each. During the training and acquisition sessions, the animals were exposed to two identical objects placed in parallel, and their exploration time was recorded. After 60 min, in the test and retention session, the animals explored 1 of the familiar objects and a novel object placed in the same position as the previous one. The exploration time for each object is registered for the determination of the Discrimination Index using the following formula:

$$\begin{gathered} Discrimination\:Index\:\left( \% \right)\: \hfill \\ = \frac{{Time\:exploring\:novel\:object\:\left( s \right)}}{{(Time\:exploring\:novel\:object\:(s)\: + \:Time\:exploring\:familiar\:object\:(s))}}\: \times \:\:100 \hfill \\ \end{gathered}$$

#### OR

On the 29th day of the experimental protocol, we performed the OR test to evaluate spatial reference memory [30]. Like the NOR test, this test is based on the natural tendency of rodents to explore a new or relocated object for longer than a familiar object. In this test, which was also executed in the OF apparatus, we completed a 5-minute training session where two identical objects placed parallel to each other were exposed to the mice to explore freely, and the exploration time for each object was documented. After 180 min, in the test session, 1 of the objects was repositioned transversally, and exploration time for each object was recorded for 5 min. The Location Index is determined by the following equation, based on the total exploration time of the object:

$$\begin{gathered} \:Location\:Index\:\left( \% \right)\: \hfill \\ = \:\frac{{Time\:exploring\:reallocated\:object\:\left( s \right)}}{{(Time\:exploring\:relocated\:object\:(s)\: + \:Time\:exploring\:familiar\:object\:(s))}}\: \times \:\:100 \hfill \\ \end{gathered}$$

#### TST

On the last day of treatment, the animals were subjected to the TST to evaluate a depressive-like phenotype [31]. For this test, the mice were suspended 50 centimeters from the ground by the distal extremity of the tail for 6 min, and the total immobility time was recorded.

## Biochemical Analysis

### Metabolic Parameters

After the behavioral tests, one cohort of animals was anesthetized with xylazine and ketamine for blood collection via cardiac puncture, which was centrifuged to obtain heparinized plasma. The total cholesterol levels in plasma were determined using a commercial enzymatic kit following the instructions provided by the manufacturer (Gold Analisa Diagnostica Ltda). On the first and last days of the experimental protocol, a capillary glucose test was conducted with blood from the tail vein. At day 0 (D0), glucose levels of both groups were measured before the beginning of the treatments. A drop of blood was collected with a test strip and tested by a glucose meter according to the instructions provided by the manufacturer (Accumed Produtos Médico Hospitalares Ltda). The results are expressed as mg/dL.

### Immunofluorescence

Another cohort of animals was anesthetized and perfused via the left ventricle with 0.9% saline solution, followed by 4% paraformaldehyde. After perfusion, whole brains were removed, immersed in 4% paraformaldehyde for 24 h, and preserved in phosphate-buffered saline (PBS) with 30% sucrose solution. Coronal sequential slices with 30 μm width of the prefrontal cortex and hippocampus were obtained using a vibrating blade microtome (Leica Biosystems VT1000S) and stored in PBS 0.1 M with 0.5% sodium azide. All immunofluorescence assays were performed according to the instructions previously described by Rodrigues et al. (2023) [15]. Initially, the slices were washed twice with PBS 0.1 M and then blocked with 1% bovine serum albumin diluted in PBS 0.1 M and 0.3% Triton X for 45 min. The slices were incubated overnight with primary antibodies for glial fibrillary acidic protein (GFAP, 1:1000 dilution, Sigma-Aldrich G3893) or ionized calcium-binding adapter molecule 1 (IBA-1, 1:500 dilution, FUJIFILM Wako 019–19741), markers for astrocytes and microglia, respectively. After 5 washes with PBS 0.1 M, the slices were incubated with secondary fluorescent antibodies (Alexa Fluor™ 488, 1:1000 dilution, Invitrogen A-11001; Alexa Fluor™ 647, 1:1000 dilution, Invitrogen A-21246) diluted in PBS 0.1 M and 0.3% Triton X for 2 h. Lastly, the slices were washed 3 times with PBS 0.1 M, mounted on glass slides with CC/mount, and covered with coverslips. For each slide representing an animal, 2 whole hippocampi from different coronal slices were imaged under 20x and 40x magnification using the Olympus^®^FV1000 confocal microscope from the UFRGS Centro de Microscopia e Microanálise. ImageJ software was used to crop the interest subregions (CA3, CA1 and dentate gyrus) and measure mean fluorescence intensity (MFI). Finally, for each hippocampus, the MFI values from the three subregions of interest were averaged, and the resulting mean for both hippocampi was used as the representative MFI for statistical analyses. For the representative panels, images from the dentate gyrus were selected, as this subregion more clearly reflected the overall pattern observed across groups.

### Western Blotting

The protein content was quantified through immunodetection in the hippocampal tissue [32]. The samples were homogenized in ice-cold lysis buffer pH 7.9: 2.5 M KCl, 10 mM Hepes, 0.6 mM EDTA, 0.1% NP 40, and 1% protease inhibitor cocktail (PIC). Equal protein concentrations (40 µg/lane of total protein, determined using a commercial kit BCA Protein Assay [Thermo Scientific Cat. no. A53225, U.S.A.]) were loaded onto NuPAGE^®^ 4%­12% Bis­Tris Gels. After electrophoresis, proteins were transferred (XCell SureLock^®^ Mini­Cell, Invitrogen Cat. no. EI0001) to nitrocellulose membranes (1 h at 50 volts in transfer buffer [48 mM Trizma, 39 mM glycine, 20% methanol, and 0.25% sodium dodecyl sulfate]). The membranes were incubated for 2 h in a blocking solution (Tris-buffered saline [TBS] plus 5% bovine serum albumin). After incubation, the membranes were incubated overnight at 4 °C in a blocking solution containing one of the following antibodies: anti-mTOR (1:1000, Cell Signaling, Cat. no. #2983), anti-pmTOR (1:1000, Cell Signaling, Cat. no. #2971), anti-AMPK (1:1000, Abcam, Cat. no. #AB80039), anti-pAMPK (1:1000, Abcam, Cat. no. #AB133448), anti-PSD95 (1:1000, Cell Signaling, Cat. no. #36233), anti-Synaptophysin (1:1000, Sigma-Aldrich, Cat. no. #SAB4200544) or anti-β­actin (1:1000, Sigma­Aldrich, Cat. no. #A4700). The blot was then washed three times for 5 min with T­TBS and incubated for 2 h in a solution containing peroxidase-­conjugated anti-­rabbit IgG (1:1000, Millipore, Cat. no. #AP132P) and peroxidase-conjugated anti-mouse IgG (1:1000, Millipore, Cat. no. #AP124P) or anti-rabbit IgG (1:1000, Millipore, Cat. no. #AP307P) and anti-mouse IgG (1:1000, Santa Cruz, Cat. no. #sc-516102). The blot was again washed four times for 5 min with T­TBS and then left in TBS. The blot was developed using a chemiluminescent ECL kit (Amersham, Oakville, Ontario; Cat. no. RPN 3004). Chemiluminescence was detected using a digital imaging system (Image Quant LAS 4000; GE Healthcare LifeSciences) and analyzed using the Image Studio Lite Software 5.2 (RRID: SCR_013715). The quantification of protein content involves calculating a ratio of intensity. The intensity of the protein of interest was measured and compared to that of anti­β-actin on the same membrane. All results are expressed as a percentage of the control.

### RT-qPCR

Hippocampal RNA extraction was performed using TRIzol^®^ Reagent (Thermo Fisher Scientific, USA) and 2-Mercaptoethanol (Sigma-Aldrich, M3148), as recommended by the manufacturer and a protocol previously described by Santos et al. (2024) [33]. RNA purity (absorbance ratio at 260 nm and 280 nm [A260/A280]) and concentration were assessed using the I-Quant device (Loccus, BR). For cDNA synthesis, 2 µg of RNA per sample was used with the High-Capacity cDNA Reverse Transcription^®^ kit (Thermo Fisher Scientific, USA). Gene-specific primers were designed using the NCBI’s Primer-BLAST tool (National Library of Medicine, USA), ensuring the absence of predicted secondary structures. Primer efficiency was evaluated to confirm the absence of nonspecific amplifications. Gene expression analysis was performed for genes encoding proteins involved in neuroplasticity, neuroinflammation, and the β-actin housekeeping gene, using the sequences in Table 1. RT-qPCR reactions were conducted in triplicate with the PowerUp™ SYBR^®^ Green Master Mix kit (Thermo Fisher Scientific, USA), following the instructions supplied by the manufacturer. Results were analyzed using the $\:{2}^{-\varDelta\:\varDelta\:CT}$ method.

**Table 1:** Primers used for RT-qPCR. Forward (F) and reverse (R) primer sequences used for RT-qPCR analysis of genes related to inflammation and synaptic plasticity

Gene	Forward sequence (5’ to 3’)	Reverse sequence (5’ to 3’)
TNF-α	ATGTCTCAGCCTCTTCTCATTC	GCTTGTCACTCGAATTTTGAGA
TLR4	CTGGGGCTCATTCACTCACTA	CTCAGACTCGGCACTTAGCA
TGF-β	ATGGTGGACCGCAACAACGC	GGCACTGCTTCCCGAATGTCTG
CD68	CTTCCCACAGGCAGCACAG	AATGATGAGAGGCAGCAAGAGG
BDNF	TGACGACGACATCACTGGCT	ACAAGTCCGCGTCCTTATGGTT
SYP	CCACCTCCTTCTCCAATCAG	CAGCAAAGACAGGGTCTCCT
TrkB	AACGGAGACTACACCCTGATGG	GCAATCACCACCACGGCATA
PSD-95	CGATTACCACTTTGTCTCCTCCC	ACGGATGAAGATGGCGATAGG
β-actin	TCAAGATCATTGCTCCTCCTGAG	ACATCTGCTGGAAGGTGGACA

### Statistical Analysis

All data were tested for normality using the Shapiro-Wilk test. Differences in glucose levels within groups were evaluated using a paired t-test. Unpaired t-tests were used for the remaining comparisons when data distribution was normal, and the Mann-Whitney test was used for non-normal data distribution. For the NOR and OR behavioral tests, the groups were compared to a hypothetical value of 50% using one-sample t-tests to analyze the chance levels. All data are presented as the mean ± standard error of the mean (SEM). Statistical significance was set at *p* < 0.05. Statistical analyses were performed using Statistica© 10 (StatSoft Inc., Tulsa, OK, USA) or GraphPad Prism© 8 (GraphPad Inc., San Diego, CA, USA). Outliers were identified using the ROUT method (Q = 5%).

## Results

### Effects of Metformin Treatment on Metabolic Alterations Related to FH

**Fig. 2:**
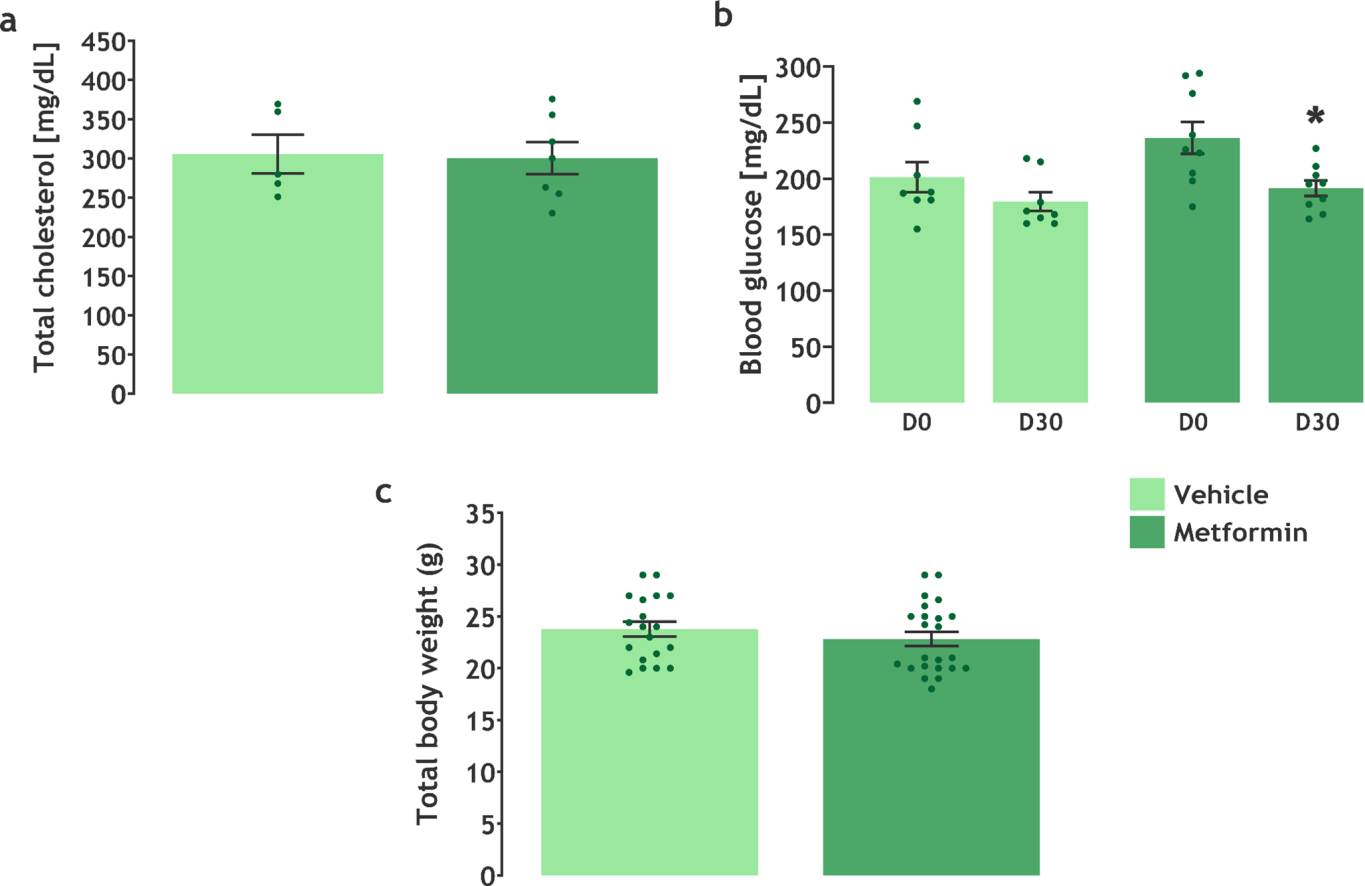
*Metformin effects on metabolic parameters of LDLr*^*−/−*^ *mice.* Plasmatic levels of **a** total cholesterol (*n* = 5–8), **b** glucose of the vehicle (left) and metformin (right) groups (*n* = 6–9), and **c** total body weight (*n* = 19–23). Data are expressed as mean ± SEM. Within-group glucose comparisons (D0 vs. D28) were analyzed using a paired t-test. Between-group comparisons for cholesterol and body weight were analyzed using an unpaired t-test. * *P* < 0.05 vs. metformin D0 (Paired t-test)

First, we evaluated the metabolic parameters of animals that received metformin treatment. Figure 2 shows the total cholesterol levels, glucose levels pre- and post-treatment, and total body weight of the LDLr^−/−^ mice. The metformin treatment did not reduce cholesterol levels (t(10) = 0.1694, *P* = 0.8689) of the FH animal model (Fig. 2a). Importantly, animals that received metformin administration showed reduced blood glucose levels on day 30 compared to day 0 (t(8) = 3.021, *P* = 0.0165) (Fig. 2b). Finally, metformin treatment did not alter the body mass (t(40) = 0.9432, *P* = 0.3512) of LDLr^−/−^ mice (Fig. 2c).

### Metformin Treatment Attenuated Behavioral Alterations in LDLR^−/−^ Mice

**Fig. 3:**
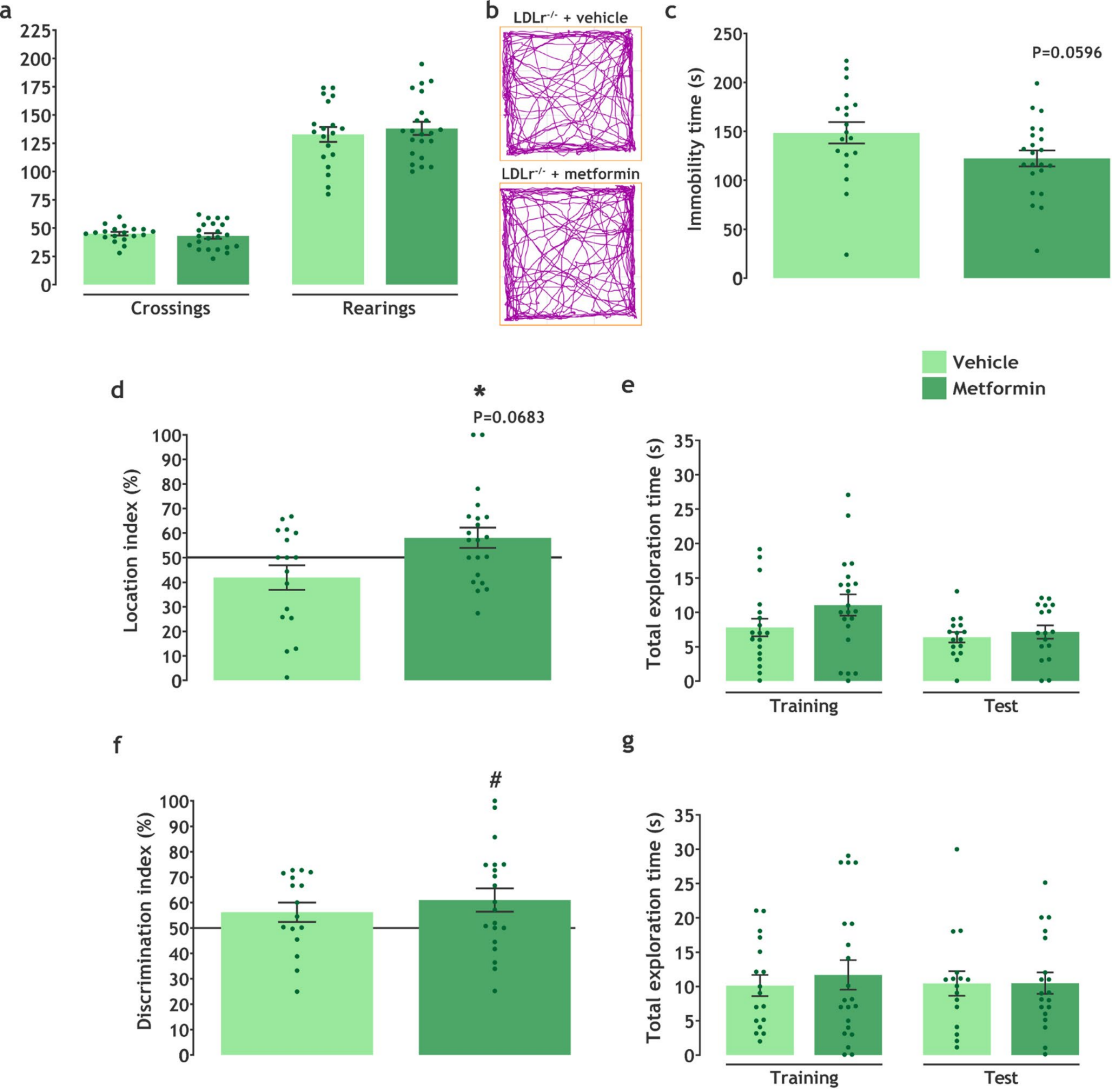
Metformin treatment improves hypercholesterolemia-induced cognitive impairment. **a** Crossings and rearings number evaluated in the OF (*n* = 19–22), **b** group-representative track plots of crossings in the OF, **c** immobility time evaluated by TST (*n* = 19–22), **d** location index evaluated by the OR (*n* = 17–21), **e** total exploration time in the OR task (*n* = 16–17), **f** discrimination index evaluated by NOR (*n* = 16–20), and **g** total exploration time in the NOR task (*n* = 16–19). Data are expressed as mean ± SEM. * *P* < 0.05 vs. vehicle (Unpaired t-test), # *P* < 0.05 vs. chance levels (50% of a new or displaced object investigation time in test trial; one sample t-test)

Analysis of the behavioral test data is shown in Fig. 3. The OF test was used to evaluate locomotor and exploratory activities by analyzing the number of crossings and rearings during the time spent in the apparatus. Metformin treatment did not change crossing (t(39) = 0.6161, *P* = 0.5414) or rearing (t(39) = 0.6397, *P* = 0.5261) numbers in LDLr^−/−^ mice (Fig. 3a, b).

To evaluate a depressive-like phenotype, the tail suspension test was performed. The unpaired t-test revealed a tendency toward a reduction in immobility time in metformin-treated animals compared to the vehicle-treated group (t(39) = 1.941, *P* = 0.0596) (Fig. 3c). Analysis of the effect size of treatment on immobility time by Cohen’s d showed a medium effect (d = 0.6).

In addition, the OR (Fig. 3d, e) and NOR (Fig. 3f, g) tests were performed to evaluate hippocampal-dependent memories. One sample t-test revealed that LDLr^−/−^ + vehicle groups showed memory and learning deficits (OR: t(16) = 1.626, *P* = 0.1235 vs. 50%; NOR: t(15) = 1.627, *P* = 0.1246 vs. 50%). Moreover, the unpaired t-test displayed that the metformin group had a higher location index than the vehicle-administered group (t(36) = 2.504, *P* = 0.0170) and a trend toward an increased location index versus chance levels (t(20) = 1.927, *P* = 0.0683 vs. 50%). Mice treated with metformin also showed an elevated discrimination index compared to chance levels by one-sample t-test (t(19) = 2.391, *P* = 0.0273 vs. 50%). These findings suggest that Metformin administration can mitigate hippocampal-associated memory deficit in the FH mice model and ameliorate their depressive phenotype.

### Metformin Decreases Hippocampal Astrogliosis in LDLR^−/−^ Mice

**Fig. 4:**
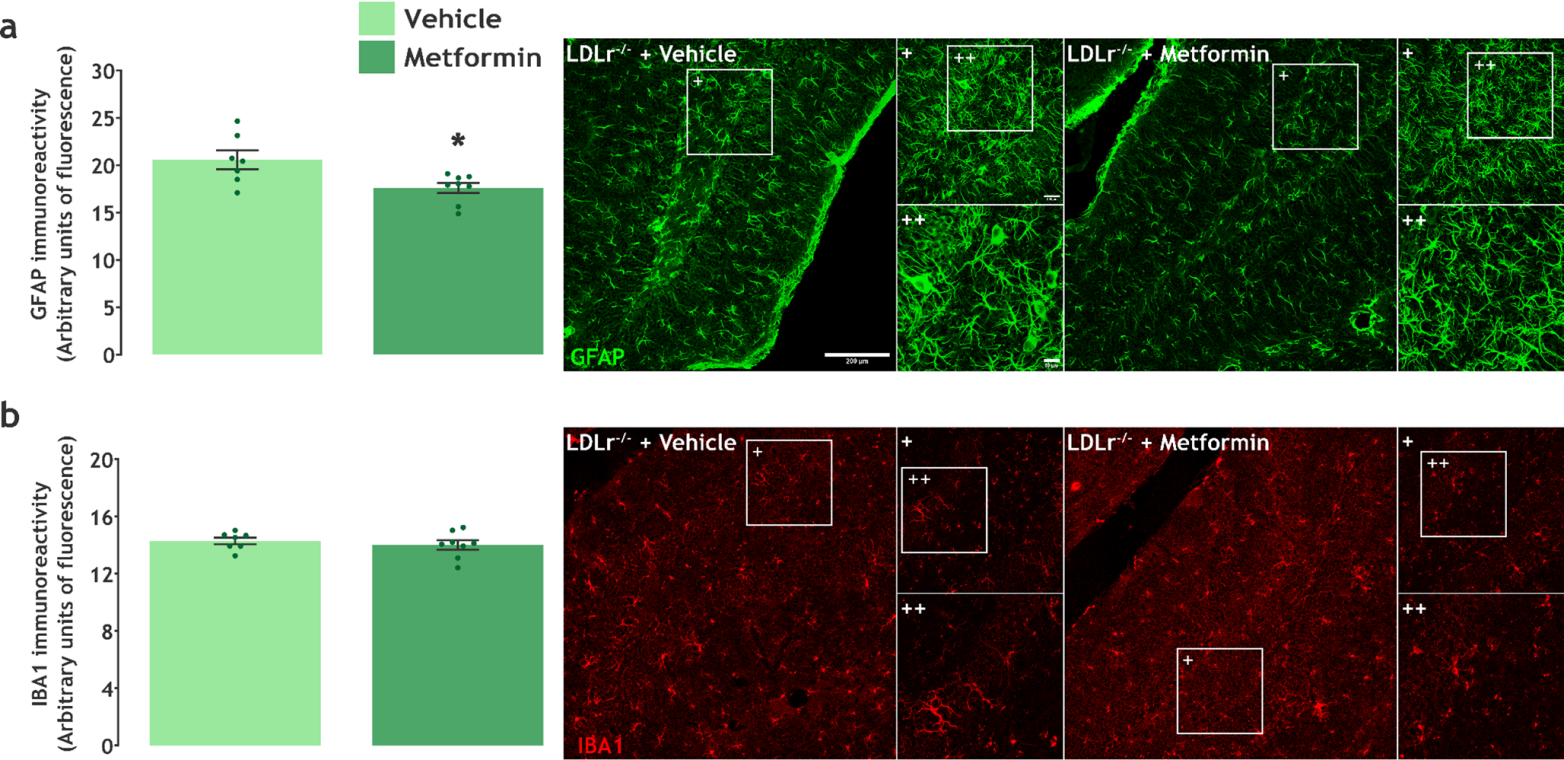
*Metformin treatment decreases GFAP immunoreactivity in the hippocampus of LDLr*^*−/−*^ *mice.* **a** GFAP immunoreactivity in total hippocampus and representative images (*n* = 7–8), and **b** IBA1 immunoreactivity in total hippocampus and representative images (*n* = 7–8). Data are expressed as mean ± SEM. * *P* < 0.05 vs. vehicle (Unpaired t-test)

Astroglial density and reactivity were evaluated by GFAP immunoreactivity in the hippocampus. Figure 4a shows representative images of the hippocampus labeled with GFAP in 20x, 40x (+), and digital zoom (++). The unpaired t-test pointed to a significant decrease (t(13) = 2.726, *P* = 0.0173) in GFAP immunoreactivity in the group treated with metformin (Fig. 4a).

To investigate the effects of metformin treatment on microglial density in the hippocampus, we performed immunofluorescence assays with IBA1. Figure 4b presents a representative panel with images of the hippocampus with IBA1 signal in 20x, 40x (+), and digital zoom (++) magnification. The unpaired t-test analysis did not detect significant statistical differences (t(13) = 0.6761, *P* = 0.5109) between groups (Fig. 4b).

### Metformin Modulates Gene Expression Related to Neuroinflammation and Synaptic Plasticity in LDLr^−/−^ Mice

**Fig. 5:**
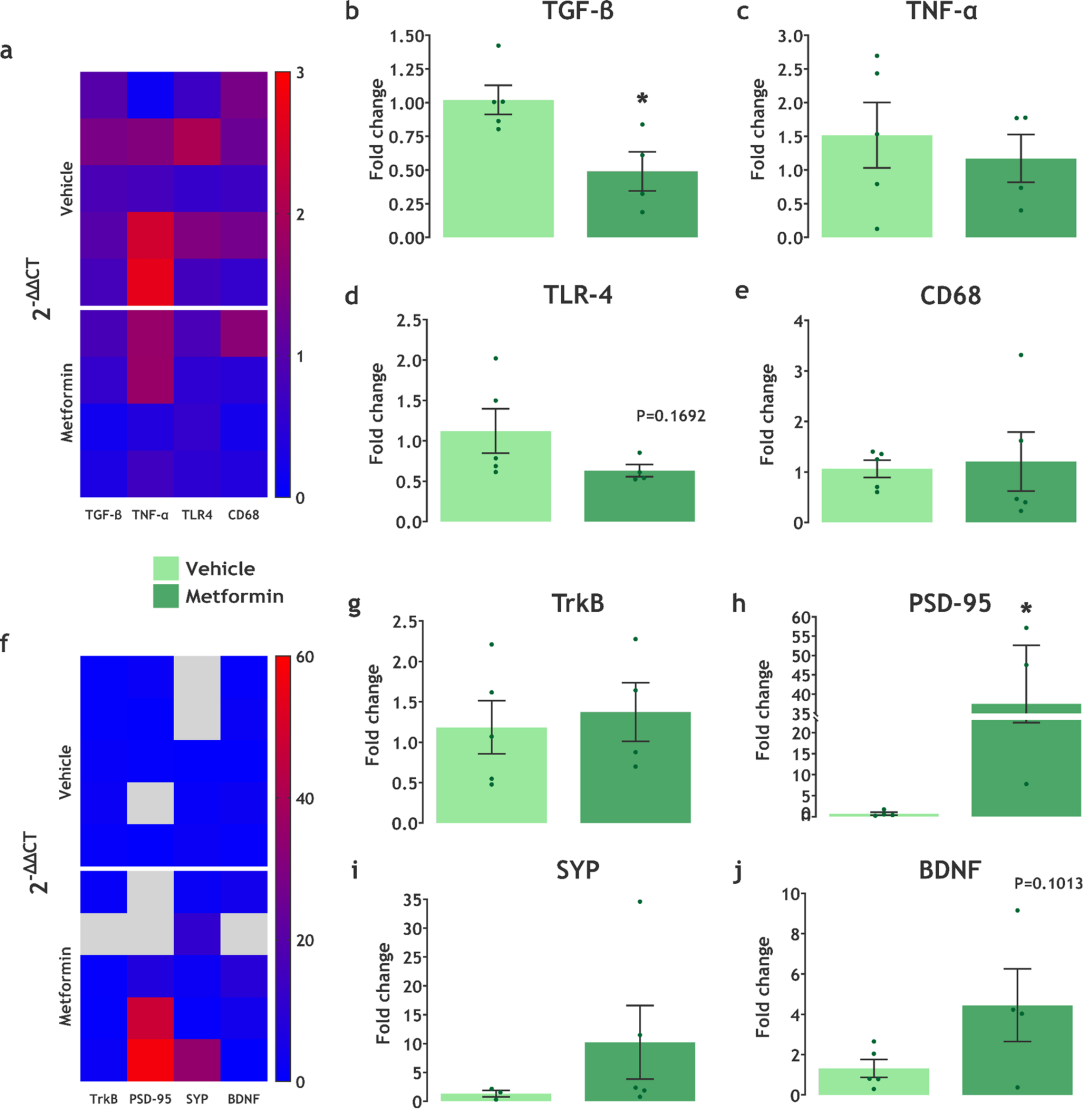
Metformin treatment modulates gene expression in the hippocampus. **a, f** Heatmap representing relative gene expression (2^⁻ΔΔCT^) on the hippocampus of LDLr^−/−^ mice treated with Metformin. Effect of vehicle (*n* = 3–5) or metformin (*n* = 3–5) administration on **b** TGF-β, **c** TNF-α, **d** TLR-4, **e** CD68, **g** TrkB, **h** PSD-95, **i** SYP, and **j** BDNF expression levels. Data are expressed as mean ± SEM. * *P* < 0.05 vs. vehicle (Unpaired t-test). Grey squares represent outlier data points

We also analyzed the gene expression of proteins associated with neuroinflammation and synaptic plasticity in the hippocampus of the animals. Our findings indicate that metformin may downregulate the expression of genes involved in inflammatory pathways, while upregulating the expression of proteins related to neuroplasticity. In particular, the hippocampus of LDLr^−/−^ treated with metformin presented a decrease in the gene expression of TGF-β (t(7) = 2.991, *P* = 0.0202) and an increase in the gene expression of PSD-95 (t(5) = 2.904, *P* = 0.0336) (Fig. 5a, b, f, h). In addition, we observed lower TLR-4 (t(7) = 1.533, *P* = 0.1692, Cohen’s d = 1.16) and higher BDNF (t(7) = 1.886, *P* = 0.1013, Cohen’s d = 0.422) expression in the hippocampus of mice treated with metformin (Fig. 5d, j), although no significant statistical difference was detected. No significant changes were observed in the expression levels of TNF-α (t(7) = 0.5474, *P* = 0.6011), CD68 (t(8) = 0.2370, *P* = 0.8186), TrkB (t(7) = 0.3857, *P* = 0.7112), or SYP (U = 3, *P* = 0.25) following metformin treatment (Fig. 5c, e, g, i).

**Fig. 6:**
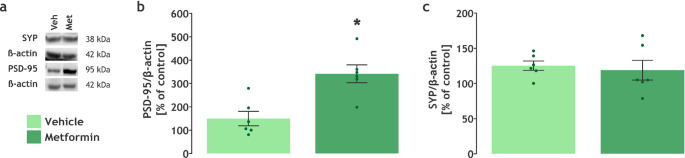
Metformin increased PSD-95 protein expression in the hippocampus. **a **Western blotting representative images. Protein content of **b** PSD-95 (*n* = 6) and **c** SYP (*n* = 6). Data are expressed as mean ± SEM. * *P* < 0.05 vs. vehicle (Unpaired t-test)

In agreement with our mRNA expression results, we also observed an increase in the PSD-95 (t(10) = 3.899, *P* = 0.0030) protein content in the hippocampus (Fig. 6a, b), but not in the SYP content (Fig. 6a, c).

### Metformin Effects are AMPK/mTOR Independent

**Fig. 7:**
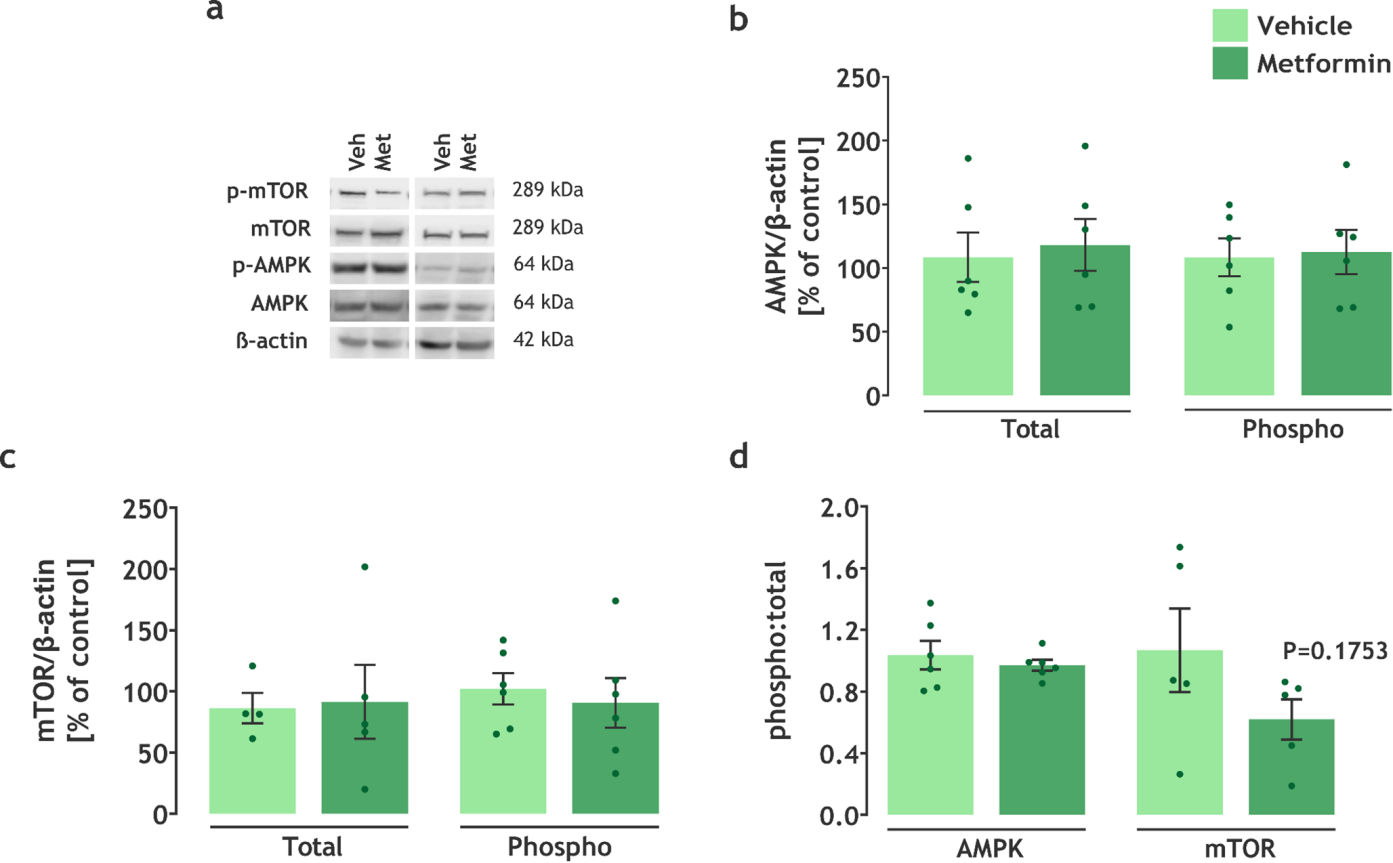
*The positive effects of metformin treatment on LDLr*^*−/−*^ *mice are independent of AMPK activation.* **a **Western blotting representative images. Protein content of **b** AMPK (*n* = 6) and **c** mTOR (*n* = 4–6), and **d** phospho: total ratio of AMPK and mTOR. Data are expressed as mean ± SEM. * *P* < 0.05 vs. vehicle (Unpaired t-test)

Considering that most of the effects of metformin treatment are associated with its modulation of the AMPK/mTOR signaling pathway, total and phosphorylated AMPK and mTOR content were assessed in the hippocampus using Western blotting. The content of both proteins, total and phosphorylated fractions, was not significantly different (AMPK: t(10) = 0.3415, *P* = 0.7398; p-AMPK: t(10) = 0.1813, *P* = 0.8597; mTOR: t(7) = 0.1434, *P* = 0.89; p-mTOR: t(10) = 0.4766, *P* = 0.6439) when comparing the groups (Fig. 7a, b, c). However, a tendency towards a lower phospho: total mTOR ratio (mTOR: t(8) = 1.487, *P* = 0.1753; AMPK: t(10) = 0.6548, *P* = 0.5274) was evidenced by the unpaired t-test (Fig. 7d) in the LDLr^−/−^ mice treated with metformin. Additionally, when analyzing the effect size of phospho: total mTOR ratio, Cohen’s d pointed to a large effect (d = 0.94).

## Discussion

In this study, we investigated the effects of metformin on metabolic, behavioral, molecular, and cellular alterations in LDLr^−/−^ mice, a well-established model of FH. Our findings reveal that, although metformin did not reduce cholesterol levels or body mass, it significantly reduced blood glucose levels after 30 days of treatment. While a random variation at baseline (D0) cannot be excluded, as both groups were untreated at that time, the consistent reduction observed after treatment supports the hypoglycemic effect of metformin. Behaviorally, metformin improved hippocampal-dependent cognitive performance in object reallocation and novel object recognition tasks and showed a trend toward alleviating depressive-like behavior. At the cellular level, metformin significantly reduced astroglial reactivity in the hippocampus, without altering microglial density. On the molecular level, metformin downregulated the expression of TGF-β and upregulated PSD-95, while also showing trends toward reduced TLR-4 and increased BDNF expression. These effects may occur independently of canonical AMPK/mTOR signaling, although a trend toward a lower phospho/total mTOR ratio was observed (d = 0.94). Collectively, these findings suggest that metformin exerts neuroprotective effects in FH, potentially by modulating neuroinflammation and synaptic plasticity through alternative molecular pathways.

Hypercholesterolemia is a well-established risk factor for the development of cardiovascular diseases, which remains the leading cause of mortality worldwide [34]. Over the past decades, hypercholesterolemia has been associated with the development of neurodegenerative diseases, such as AD [1]. More recently, high plasmatic LDL-cholesterol levels during midlife have been recognized as a significant risk factor for dementia [6]. The most common form of genetic hypercholesterolemia, FH, is typically caused by mutations that impair LDLr function and reduce LDL clearance from the circulation [35, 36]. Notably, a clinical study demonstrated that FH individuals can present executive function impairment as early as 18 to 40 years old [7]. Complementing these findings, experimental studies using LDLr^⁻/⁻^ mice, a widely used model of FH, have demonstrated hippocampal neuroinflammation [37], astrogliosis and blood-brain barrier disruption [12], depressive-like behavior [11], and memory deficits as early as 3 months of age [38]. These observations underscore the urgent need for effective therapeutic strategies to prevent or mitigate the neurological consequences of FH. In this context, we investigated the effects of metformin administration on metabolic, behavioral, and glial alterations in LDLr^−/−^ mice.

Metformin is a first-line oral antidiabetic drug widely used to improve insulin sensitivity and regulate glucose metabolism, particularly in individuals with type 2 diabetes [39, 40]. Beyond its effects on blood glucose levels, metformin has also been associated with cardiovascular benefits, including lipid-lowering properties [41, 42]. Human studies have shown that metformin reduces the risk of cardiovascular disease in diabetic patients by lowering circulating LDL cholesterol levels and reducing atherosclerotic plaque thickness [43, 44]. Both acute and chronic metformin treatment tend to reduce total and LDL cholesterol levels, while having minimal effects on plasma triglycerides and HDL cholesterol [41, 42, 45, 46]. These effects are supported by preclinical data. In hyperglycemic mice exposed to an atherogenic diet, metformin reduced atherosclerotic lesion formation by nearly 60% [47]. Likewise, in ApoE^−/−^ mice fed a high-fat diet, metformin treatment reduced atherosclerotic burden and was associated with lower body weight [48–51]. However, findings in non-diabetic hypercholesterolemic models have been more variable. Some studies report that metformin does not significantly affect circulating lipid levels in these models [24, 52]. Consistent with these reports, our study found that metformin did not reduce total cholesterol or body weight in FH mice. Nevertheless, it significantly lowered blood glucose levels after 30 days of treatment, suggesting a selective metabolic benefit that could still support vascular and cognitive health.

As previously demonstrated, LDLr^⁻/⁻^ mice between 3 and 5 months of age already exhibit significant memory impairments [13]. In the present study, metformin treatment improved learning and spatial memory deficits in these hypercholesterolemic mice. Animals receiving metformin spent more time exploring novel or reallocated objects in recognition-based memory tasks compared to vehicle-treated controls. However, while a group difference was observed in the OR, metformin did not fully reverse memory impairment: the location index did not significantly differ from the hypothetical 50% chance level. These findings are in line with previous studies demonstrating that metformin can improve cognitive performance in various models of neurological dysfunction, including AD, obesity, and diabetes [25, 53–55]. For example, genetically diabetic mice treated with 200 mg/Kg of metformin for six weeks showed enhanced learning and memory performance in the Morris water maze [54]. Likewise, rats fed a high-fat diet recovered learning behavior in the same task following a 12-week treatment with 30 mg/Kg of metformin [55]. Taken together, our results indicate that metformin ameliorated, but not fully reversed, the cognitive and brain dysfunction observed in LDLr^−/−^ mice, consistent with prior literature aforementioned.

Another behavioral alteration frequently reported in animal models of hypercholesterolemia is a depressive-like phenotype [11, 56]. Increased immobility in the forced swim test and TST, hallmarks of depressive-like behavior, has been consistently observed in mice subjected to diet-induced hypercholesterolemia [4, 56]. Similarly, Engel et al. (2016) demonstrated that LDLr^−/−^ mice exhibit depressive-like behavior across multiple paradigms, including the TST, sucrose preference, and sucrose splash tests [11]. In the present study, metformin treatment tended to reduce immobility time in LDLr^−/−^ mice, suggesting a potential improvement in depressive-like behavior. This finding aligns with clinical evidence; for instance, Guo et al. (2014) reported that metformin treatment in diabetic patients with comorbid depression led to a reduction in depressive symptoms [57]. Moreover, experimental data from C57BL/6J male mice fed a high-fat diet showed that chronic administration of metformin (300 mg/Kg) produced antidepressant-like effects, further supporting the psychotropic potential of the drug in metabolically compromised conditions [58]. Collectively, these findings reinforce the hypothesis that metformin may grant mood-stabilizing or antidepressant-like benefits in the context of metabolic disturbances, such as those associated with FH.

Neuroinflammation is increasingly recognized as a central mechanism linking hypercholesterolemia to the pathogenesis of neurodegenerative diseases, including AD [12, 13, 59]. Clinical studies have shown that elevated levels of pro-inflammatory cytokines correlate with cognitive impairment in AD patients and elderly individuals [60–62], while anti-inflammatory interventions in animal models of AD improve memory outcomes [63, 64]. In hypercholesterolemic conditions, both systemic and cerebral inflammation have been documented [37, 65, 66], with glial reactivity—particularly of astrocytes and microglia—playing a key role in the propagation of neuroinflammatory responses [12, 67]. Astrocytes, which normally regulate metabolism, support neurons, and facilitate synaptogenesis, can be activated via NF-κB-mediated inflammatory pathways [68, 69]. In this inflammatory milieu, activated astrocytes contribute to the production of reactive nitrogen species and other inflammatory mediators [70, 71] and exhibit increased expression of GFAP, which is a key cytoskeletal protein that serves as a marker of astrocytic reactivity [72]. Similarly, metformin has been shown to attenuate astrocyte reactivity in models of diabetes [73, 74], Parkinson’s disease [75], and fetal alcohol syndrome [76], highlighting its potential as a therapeutic modulator of neuroinflammation.

Microglia play a central role in the innate immune response of the central nervous system, acting as key regulators of neuroinflammation and homeostasis [77]. In addition to its immune surveillance function, microglia contribute to neurogenesis, synaptic pruning, and the secretion of pro-inflammatory cytokines in response to pathological stimuli [78, 79]. Their activation profile is highly context-dependent and varies according to the nature, intensity, and chronicity of the triggering insult [80]. In a previous study from our group, we demonstrated that LDLr^⁻/⁻^ mice exhibit increased hippocampal IBA1 immunoreactivity at 3, 6, and 14 months of age, indicative of heightened microglial activation compared to wild-type controls [15]. In the present study, however, metformin treatment did not reduce hippocampal microglial density in LDLr^⁻/⁻^ mice. This finding contrasts with previous reports showing that metformin attenuates microgliosis in models of high-fat diet-induced neuroinflammation. For example, Ma et al. (2021) reported a significant reduction in IBA1-positive microglia in the hippocampus of 8-month-old C57BL/6J male mice following 3 months of treatment with 250 mg/Kg/day of metformin [81]. Importantly, discrepancies in the observed effects may stem from differences in treatment duration, animal age, or disease context. Supporting this, Kodali et al. (2021) found that metformin administration for 10 weeks in aged mice did not reduce overall IBA1 expression in the hippocampus but did diminish the tendency of microglia to form pro-inflammatory clusters [23]. These findings suggest that the effects of metformin on microglial activation may depend on the stage of disease progression and specific microglial phenotypes rather than simply reducing cell density.

Although we did not observe changes in microglial density following metformin treatment in LDLr^⁻/⁻^ mice, we further explored potential molecular shifts in neuroinflammatory signaling and neuroplasticity by analyzing hippocampal gene expression profiles. This approach provides insights into modulatory effects of metformin beyond its effects on microglial morphology or density. Notably, our results demonstrated a significant downregulation of TGF-β gene expression in the hippocampus of metformin-treated LDLr^⁻/⁻^ mice. Importantly, this finding should be interpreted in light of the context-dependent actions of TGF-β in the central nervous system. While TGF-β can exert anti-inflammatory and neuroprotective effects under physiological or acute stress conditions, persistent upregulation of this pathway has been linked to chronic glial activation, synaptic dysfunction, and neurodegenerative processes [82]. Therefore, rather than suggesting that reduced TGF-β expression is inherently beneficial, our data may indicate a shift away from a chronically reactive glial state commonly associated with LDLr deficiency [15]. This interpretation is consistent with previous studies showing that metformin can suppress TGF-β1 signaling in peripheral and central inflammatory models. For instance, in a chronic colitis mouse model, metformin reduced TGF-β1 expression and Smad3 phosphorylation, mitigating inflammation and fibrosis [83]. Moreover, Xiao et al. (2022) showed that metformin downregulated INHBA, a key ligand of TGF-β signaling, in colorectal cancer cells, thereby inhibiting PI3K/Akt activation and cell proliferation [84]. Together, these data support the hypothesis that metformin may reduce TGF-β–mediated glial activation in neuroinflammatory contexts, contributing to its neuroprotective effects in LDLr^−/−^ mice. It is important to note, however, that these findings are based on transcriptional changes. A limitation of the present study is the lack of protein-level validation for TGF-β. Therefore, while our data suggest metformin may modulate this pathway, further research is needed to confirm if these mRNA changes result in altered TGF-β protein expression and signaling.

In parallel, metformin treatment increased the gene expression and protein content of PSD-95, a key synaptic scaffolding protein associated with synaptic stability and plasticity, indicating a potential enhancement of hippocampal synaptic function. Given the established role of PSD-95 in anchoring NMDA and AMPA receptors and supporting synaptic strength [85], its upregulation likely contributes to the observed improvements in hippocampal-dependent memory. This finding aligns with prior evidence showing that, in a rat sepsis model, metformin restored PSD‑95 levels along with other synaptic markers disrupted by systemic inflammation [86]. Because learning and memory depend on changes in synaptic efficacy, including the strengthening of glutamatergic transmission, increased PSD-95 can enhance synaptic stability, receptor retention at the synapse, and overall efficiency of postsynaptic signaling [87, 88]. These mechanisms are directly relevant for the encoding and retrieval of object recognition and discrimination, which underlie performance in the NOR and OR tasks [87, 88].

Importantly, our research group has previously demonstrated that LDLr^−/−^ mice exhibit reduced hippocampal PSD-95 immunocontent at 3 months of age compared to age-matched C57BL/6 WT mice [15]. This reduction was associated with impaired performance in the NOR test, as untreated LDLr^−/−^ animals explored the familiar object more than the novel object during the test session [12]. These findings support a functional association between lower PSD-95 levels and hippocampus-dependent memory deficits. In this context, the upregulation of PSD-95 observed in the present study following metformin treatment provides a biologically plausible mechanism that may contribute to the improved behavioral performance observed in LDLr^−/−^ mice. This connection strengthens the link between our molecular results and the behavioral improvements observed in the NOR and OR tasks.

Similarly, in APP/PS1 transgenic mice, metformin improved synaptic integrity and increased PSD-95 expression [89]. However, in contrast, Cho et al. (2024) reported that long-term metformin administration (1–2 years) in 3xTg-AD mice resulted in decreased PSD-95 expression, suggesting model- and time-dependent effects [90]. Together, these findings reinforce the notion that metformin supports hippocampal synaptic plasticity, which may underlie the cognitive improvements observed in our LDLr^−/−^ mouse model.

While changes in TLR4 and BDNF expression did not reach statistical significance, the moderate-to-large effect sizes observed (Cohen’s d = 1.16 for TLR4 and d = 0.42 for BDNF) indicate potential biological trends toward reduced innate immune activation and enhanced neurotrophic support. Although non-significant, the observed trend toward increased BDNF expression aligns with prior reports demonstrating metformin-induced upregulation of BDNF in models of depression and cognitive impairment [91, 92]. These molecular shifts complement our behavioral findings, which showed improved memory performance and reduced depressive-like behavior in metformin-treated animals. Altogether, the data supports the hypothesis that metformin may exert neuroprotective effects in FH by modulating inflammatory signaling and enhancing synaptic resilience.

**Fig. 8:**
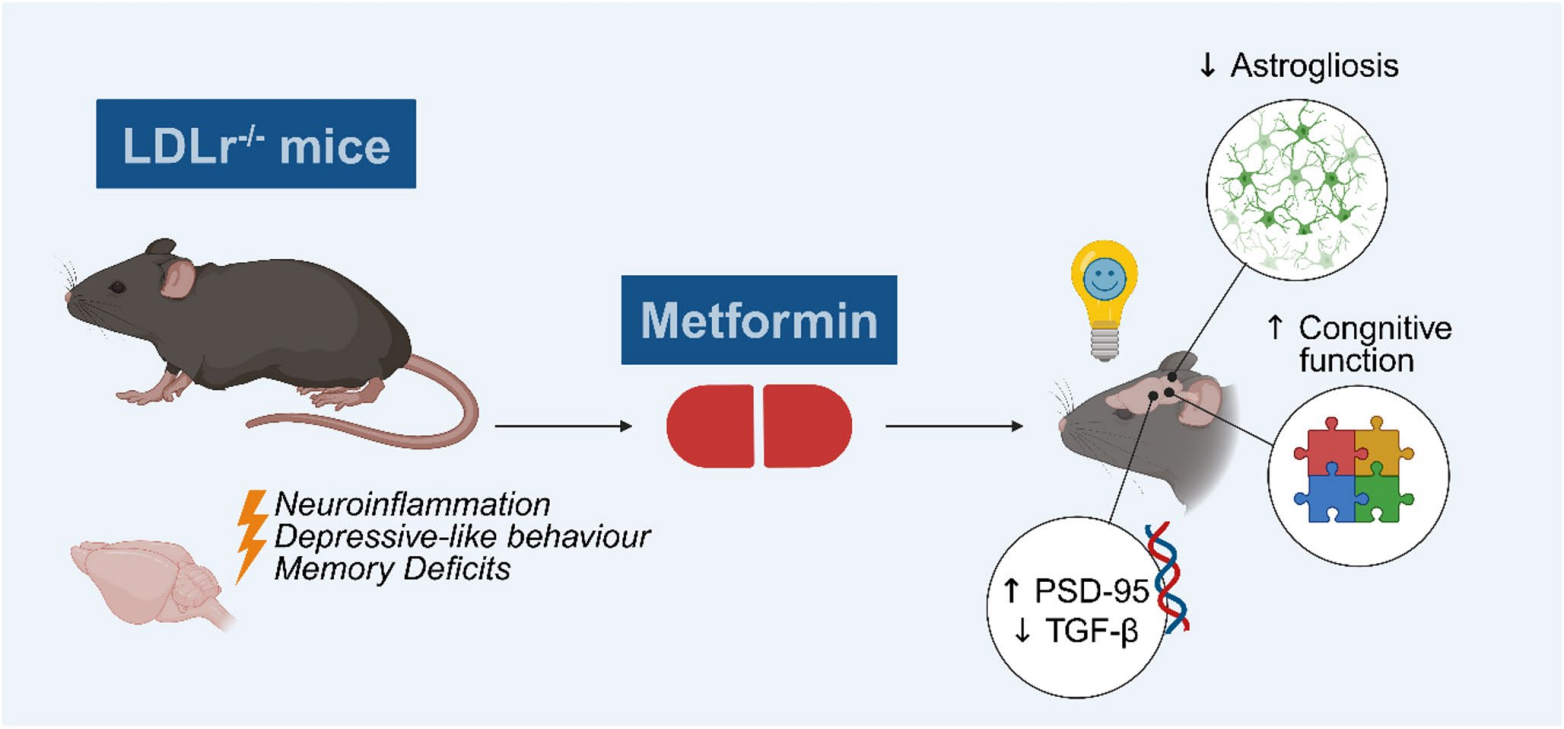
Key findings. LDLr^−/−^ mice present neuroinflammation and behavioral alterations such as a depressive-like phenotype and memory impairment. Treatment with metformin reduced hippocampal astrogliosis, ameliorated cognitive function, increased gene expression of PSD-95 and decreased gene expression of TGF-β in the hippocampus

Interestingly, we observed a trend toward a reduced phospho/total mTOR ratio in the hippocampus of metformin-treated LDLr^−/−^ mice, with a large effect size, suggesting a potential downregulation of mTOR activity. This observation is consistent with previous findings indicating that metformin can suppress mTOR signaling, most commonly via activation of AMPK, a key energy sensor that negatively regulates the mTOR pathway [20, 93]. Given that phosphorylated mTOR, particularly within the mTORC1 complex, represents its active state, the observed reduction in phosphorylation likely reflects decreased mTORC1 activity. In the central nervous system, inhibition of mTORC1 has been associated with multiple neuroprotective mechanisms, including enhanced autophagy, reduced neuroinflammation, and improved synaptic plasticity. Indeed, in animal models, pharmacological inhibition of mTOR with rapamycin has been shown to rescue behavioral deficits, including depressive-like behavior and cognitive deficits [94, 95]. Of particular relevance, rapamycin-mediated mTOR inhibition has been shown to restore neurovascular coupling and memory performance in AD mouse models by reversing both nitric oxide synthase-dependent and independent cerebrovascular deficits [96]. Although our data indicates that metformin effects were not accompanied by significant modulation of the AMPK/mTOR pathway in the hippocampus, this does not exclude the involvement of alternative mechanisms. Metformin is known to influence several targets beyond AMPK, including inhibition of mitochondrial complex I, leading to reduced oxidative stress and altered energy metabolism [97]. Furthermore, AMPK activation can be cell-type-specific, and subtle changes in astrocytes or microglia may not be detected when assessing whole-tissue homogenates. In this regard, the observed reduction in GFAP immunoreactivity, which suggests that metformin may act preferentially on astrocytes. Another possible explanation involves indirect effects through improved peripheral insulin sensitivity and systemic metabolic regulation, which could secondarily attenuate neuroinflammatory signaling [98]. Future studies dissecting cell-type-specific pathways will be valuable to clarify these mechanisms. Therefore, the trend toward mTOR inhibition observed here may reflect a cumulative outcome of multiple upstream regulatory inputs, potentially amplified by the metabolic disturbances inherent to LDLr^−/−^ mice. Collectively, our findings suggest that metformin may contribute to neuroprotective effects in hypercholesterolemic conditions (Fig. 8), at least in part, through modulating the AMPK/mTOR axis. This pathway is of particular interest in the context of aging and neurodegeneration, as its dysregulation has been implicated in both processes. In line with this, large-scale clinical trials such as “Targeting Aging with Metformin” and “Investigation of Metformin in Pre-Diabetes on Atherosclerotic Cardiovascular OuTcomes” are currently investigating the broader systemic and neurological benefits of metformin in non-diabetic populations [99]. Our results contribute to this growing body of evidence by highlighting potential central mechanisms through which metformin may exert protective actions on brain health.

It is important to acknowledge some limitations of this study. First, the absence of a concurrent wild-type control group restricts our ability to contextualize the magnitude of the observed effects relative to baseline physiology. However, this choice is also consistent with efforts to reduce animal use, given that the LDLr^−/−^ model is well characterized in the literature. Another limitation concerns the relatively small sample size used for the molecular analyses, which may have reduced the statistical power to detect more subtle changes in targets that did not reach significance, such as TLR-4 and BDNF. Finally, a limitation of our study is that the analysis of several molecular markers was conducted at the mRNA level only, without corresponding protein-level validation. Future studies will be important to more fully elucidate the mechanisms underlying the effects of metformin in LDLr^−/−^ mice [100].

## Conclusion

In conclusion, our findings suggest that metformin improves cognitive and mood-related behaviors in LDLr^−/−^ mice, accompanied by reduced hippocampal astrogliosis and transcriptional evidence of decreased neuroinflammatory signaling and enhanced synaptic plasticity. While canonical metabolic pathways like AMPK and mTOR were not significantly modulated in our dataset, our data point to additional mechanisms, including suppression of TGF-β and upregulation of PSD-95. These results add to the growing body of evidence supporting the pleiotropic benefits of metformin on brain health and support further investigation into its potential use in FH and other hypercholesterolemic conditions.

## Data Availability

The data that support this study are available from the corresponding author upon reasonable request.
